# Optimizing diagnostic algorithms to advance Hepatitis C elimination in Italy: A cost effectiveness evaluation

**DOI:** 10.1111/liv.15070

**Published:** 2021-10-08

**Authors:** Andrea Marcellusi, Francesco Saverio Mennini, Murad Ruf, Claudio Galli, Alessio Aghemo, Maurizia R. Brunetto, Sergio Babudieri, Antonio Craxi, Massimo Andreoni, Loreta A. Kondili

**Affiliations:** ^1^ Economic Evaluation and HTA (EEHTA) CEIS Faculty of Economics University of Rome “Tor Vergata” Rome Italy; ^2^ Institute of Leadership and Management in Health Kingston Business School Kingston University London UK; ^3^ Public Health, Medical Affairs Gilead Science London UK; ^4^ Global Medical and Scientific Affairs Core Laboratory, Abbott Rome Italy; ^5^ Department of Biomedical Sciences Humanitas University Pieve Emanuele Italy; ^6^ Division of Internal Medicine and Hepatology Humanitas Research Hospital IRCCS Rozzano Italy; ^7^ Internal Medicine Department of Clinical and Experimental Medicine University of Pisa Pisa Italy; ^8^ Hepatology Unit and Laboratory of Molecular Genetics and Pathology of Hepatitis Viruses University Hospital of Pisa Pisa Italy; ^9^ Infectious and Tropical Disease Unit Department of Medical Surgical and Experimental Sciences University of Sassari Sassari Italy; ^10^ Gastroenterology and Hepatology Unit Department of Internal Medicine and Medical Specialties “PROMISE” University of Palermo Palermo Italy; ^11^ Department of Systems Medicine University of Rome “Tor Vergata” Rome Italy; ^12^ Infectious Diseases Clinic University Hospital “Tor Vergata” Rome Italy; ^13^ Center for Global Health Istituto Superiore di Sanità Rome Italy

**Keywords:** cost‐effectiveness, HCV chronic infection, screening, WHO targets

## Abstract

**Objectives:**

Optimized diagnostic algorithms to detect active infections are crucial to achieving HCV elimination. We evaluated the cost effectiveness and sustainability of different algorithms for HCV active infection diagnosis, in a context of a high endemic country for HCV infection.

**Methods:**

A Markov disease progression model, simulating six diagnostic algorithms in the birth cohort 1969‐1989 over a 10‐year horizon from a healthcare perspective was used. Conventionally diagnosis of active HCV infection is through detection of antibodies (HCV‐Ab) detection followed by HCV‐RNA or HCV core antigen (HCV‐Ag) confirmatory testing either on a second sample or by same sample reflex testing. The undiagnosed and unconfirmed rates were evaluated by assays false negative estimates and each algorithm patients’ drop‐off. Age, liver disease stages distribution, liver disease stage costs, treatment effectiveness and costs were used to evaluate the quality‐adjusted life‐years (QALYs) and the incremental cost‐effectiveness ratios (ICER).

**Results:**

The reference option was Rapid HCV‐Ab followed by second sample HCV‐Ag testing which produced the lowest QALYs (866,835 QALYs). The highest gains in health (QALYs=974,458) was obtained by HCV‐RNA reflex testing which produced a high cost‐effective ICER (€891/QALY). Reflex testing (same sample‐single visit) vs two patients’ visits algorithms, yielded the highest QALYs and high cost‐effective ICERs (€566 and €635/QALY for HCV‐Ag and HCV‐RNA, respectively), confirmed in 99.9% of the 5,000 probabilistic simulations.

**Conclusions:**

Our data confirm, by a cost effectiveness point of view, the EASL and WHO clinical practice guidelines recommending HCV reflex testing as most cost effective diagnostic option vs other diagnostic pathways.

**Abbreviations**
Ab: Antibodies‐
Ag: Antigen
CEAC: cost‐effectiveness acceptability curve
DAAs: Direct Acting Antivirals
DSA: deterministic sensitivity analyses
F: Fibrosis stage
HCV: Hepatitis C virus
HCV‐Ag: HCV core antigen
ICER: incremental cost‐effectiveness ratio
MIN–MAX: Minimum‐Maximum
PSA: probabilistic sensitivity analyses
QALY: quality‐adjusted life‐years
RNA: Ribonucleic Acid
SoC: Standard of Care
WHO: World Health Organization
WTP: willingness‐to‐pay

**Lay Summary**

Hepatitis C virus (HCV) is a leading cause of liver‐related morbidity and mortality worldwide. The limitation of HCV therapy is the identification of available patients to treat because chronic infection often remains asymptomatic thus undiagnosed. The data reported in this study are of importance in that they demonstrate the value for money profiles of several diagnostic algorithms used for HCV screening in the general population. In this study, the most effective diagnostic algorithm for HCV screening in terms of value for money spent was reflex testing, which is confirmatory testing of possible infection is done on the sample blood sample not requiring a second patient visit to healthcare. Based on these results, reflex testing should become the standard of care for HCV screening in each epidemiological peculiarities of HCV infection in that it can support increases in diagnosis and subsequent treatment of infected patients necessary for achieving elimination.

## 1 INTRODUCTION

Chronic viral hepatitis C is a major public health problem. Achieving the WHO’s Global Health Sector Strategy (GHSS) goals for the elimination of Hepatitis C virus (HCV) by 2030 has reinvigorated public health initiatives aimed at identifying patients with HCV related disease.^1^

Italy is one of the countries with the greatest burden of HCV in Western Europe.^2^, ^3^ Up to now, more than 220,000 patients have been treated with Direct Acting Antivirals (DAAs), which are estimated as 40%‐60% of infected individuals, the remaining are estimated at least 280,000 individuals mostly unaware of their HCV active infection.^4^, ^5^, ^6^ In order to achieve HCV elimination by 2030 Italy, like many other countries, will need to succeed in tackling the undiagnosed proportion of infected individuals. The Italian Governative *“Milleproroghe Decree”,* through an amendment approved in March 2020, has allocated €71.5 million for the period 2020‐2022 to introduce free‐of‐charge screening for the general population born between 1969 and 1989, as well as all individuals at public specialist facilities for drug addiction and prisons. Although the screening budget has been established, optimisation along the entire patient pathway is necessary to achieve elimination by 2030.^7^ Crucially, high enough coverage level for treatment in the first instance also depends on optimized diagnostic pathways to confirm active infection. In order to realize an effective screening strategy and to overcome challenges on the adherence, simple diagnostic paths to avoid losing substantial shares of patients with active infections has been proposed by the scientific community.^8^, ^9^, ^10^, ^11^

The aim of this study was to evaluate the cost‐effectiveness of different diagnostic algorithms for active HCV infection including conventional two steps algorithms and same sample reflex testing (single step) combined with modelling treatment impacts and disease progression in order to provide for a complete overview of diagnostic costs and benefits.

## 2 METHODS

The primary outcome measure of screening effectiveness was the number of active infections diagnosed. An adapted multicohort Markov model (Figure S1) capturing multiple states of morbidity and mortality was used to evaluate the HCV disease progression and related costs for linked‐to‐care patients vs those not linked over a 10‐year time horizon (years 2020‐2030).^12^, ^13^

We compared strategies in terms of the total costs of screening according to each diagnostic algorithm and treatment costs of active HCV infection vs the disease costs of those not diagnosed over time. We considered the Italian general population birth cohort (1969‐1989) screening.

The model inputs are shown in Tables 1 and 2.

**TABLE 1:** Decision Tree epidemiological parameters

	Base‐case	Min	Max	Sources
Population born 1969‐1989 *	16,978,388	12,733,791	21,222,985	ISTAT. Resident Population, By Age. 2020. dati.istat.it. Accessed 17/10/2020.
Screening coverage rate	70%	53%	88%	Assumption
Number of prevalent undiagnosed HCV patient	115,000	86,250	143,750	Estimations from [14]
% of prevalent undiagnosed HCV patient	0.7%	0.5%	0.8%	Calculation
1.a) Rapid Ab assay +confirmation (RNA)				
Ab HCV+/HCV RNA‐	0.30%	0.24%	0.36%	[15]
Unconfirmed	45.00%	36.00%	54.00%	[16]
Undiagnosed	7.50%	6.00%	9.00%	False Negative 1st and 2nd line test (7% for anti‐HCV [17, 18]; 0.5% for HCV‐RNA – assumption)
1.b) Rapid Ab assay +confirmation (Ag)				
Ab HCV+/HCV A‐g‐	0.30%	0.24%	0.36%	[15]
Unconfirmed	45.00%	36.00%	54.00%	[16]
Undiagnosed	10.50%	8.40%	12.60%	False Negative 1st and 2nd line test (7% for anti‐HCV [17, 18]; 3.5% for HCV‐Ag [19])
2.a) Lab‐based Ab assay +confirmation (RNA) with second sample taken				
Ab HCV+/HCV RNA‐	0.30%	0.24%	0.36%	[15]
Unconfirmed	45.00%	36.00%	54.00%	[16]
Undiagnosed	2.50%	2.00%	3.00%	False Negative 1st and 2nd line test (2% for anti‐HCV [20]; 0.5% for HCV‐RNA – assumption)
2.b) Lab‐based Ab assay +confirmation (Ag) with second sample taken				
Ab HCV+/HCV Ag‐	0.30%	0.24%	0.36%	[15]
Unconfirmed	45.00%	36.00%	54.00%	[16]
Undiagnosed	5.50%	4.40%	6.60%	False Negative 1st and 2nd line test (2% for anti‐HCV [20]; 3.5% for HCV‐Ag [21])
3.a) Lab‐based Ab assay +confirmation (RNA) reflex testing				
Ab HCV+/ HCV RNA‐	0.30%	0.24%	0.36%	[15]
Unconfirmed	17.00%	13.60%	20.40%	[16]
Undiagnosed	2.50%	2.00%	3.00%	False Negative 1st and 2nd line test (2% for anti‐HCV [20]; 0.5% for HCV‐RNA – assumption)
3.b) Lab‐based Ab assay +confirmation (Ag) reflex testing				
Ab HCV+/ HCVAg‐	0.30%	0.24%	0.36%	(15)
Unconfirmed	17.00%	13.60%	20.40%	(16)
Undiagnosed	5.50%	4.40%	6.60%	False Negative 1st and 2nd line test (2% for anti‐HCV [20]; 3.5% for HCV‐Ag [21])
Fibrosis distribution of patients that are undiagnosed				
F0‐F2	75%	56%	94%	[5, 14]
F3	20%	15%	25%	[5, 14]
F4	5%	4%	6%	[5, 14]
DC+HCC	0%	0%	0%	[5, 14]
Fibrosis distribution of patients that are Unconfirmed/Unlinked to care				
F0‐F2	75%	56%	94%	[5, 14], Assumption
F3	20%	15%	25%	[5, 14]
F4	5%	4%	6%	[5, 14]
DC+HCC	0%	0%	0%	[5, 14], Assumption
Fibrosis distribution of patients that will be diagnosed by screening				
F0‐F2	70%	53%	88%	[5, 14]
F3	10%	8%	13%	[5, 14]
F4	15%	11%	19%	[5, 14]
DC+HCC	5%	4%	6%	[5, 14]
Years without diagnosis for Undiagnosed / Unconfirmed patients				
F0‐F2	10	7.5	12.5	Assumption
F3	4	3	5	Assumption
F4	1	0.75	1.25	Assumption
DC+HCC	1	0.75	1.25	Assumption

Note

“Unconfirmed” cases were defined as HCV‐Ab positive individuals who did not reattend for confirmatory testing, thus are not linked to care.

“Undiagnosed” cases were defined as having active HCV infection but with HCV‐Ab false negative results, or false negative confirmation test following an anti‐HCV positive test result

Abbreviations: Ab, Antibodies; Ag, Antigen; DC, Decompensated Cirrhosis; HCC, Hepatocellular Carcinoma; HCV, Hepatitis C Virus; RNA, Ribonucleic Acid.

* HCV screening is offered free of charge in individuals from general population born between 1969 and 1989.

**TABLE 2:** Decision tree and markov model parameters

	Decision tree				
First Line Test	Base‐case	Min	Max	SE	Source
Ab Essay	€ 5	€ 4	€ 6	€ 1	Law Reimbursed
Administration Rapid Ab Essay	€ 3	€ 2.4	€ 3.6	€ 1	Assumption
Administration Ab Essay (Second Sample or Reflex)	€ 5	€ 4	€ 6	€ 1	Italian Ministerial Decree (18 October 2012)
RNA confirmatory test	€ 68.35	€ 54.68	€ 82.02	€ 10	Italian Ministerial Decree (18 October 2012)
Ag confirmatory test	€ 15.85	€ 12.68	€ 19.02	€ 13	Assumption

	Markov model				
Cost of treatment	Base‐case	Min	Max	SE	Source
Treatment Cost	€ 6,000.00	€ 4,800.00	€ 7,200.00	€ 1,000.00	Assumption by local experts
Other direct medical costs	Base‐case	Min	Max	SE	Source
F0	€ 234	€ 176	€ 292	€ 25	[13]
F1	€ 234	€ 176	€ 292	€ 25	[13]
F2	€ 234	€ 176	€ 292	€ 25	[13]
F3	€ 617	€ 292	€ 942	€ 139	[13]
F4	€ 876	€ 397	€ 1,354	€ 205	[13]
DC	€ 6,627	€ 4,385	€ 8,868	€ 962	[13]
HCC	€ 12,896	€ 5,792	€ 20,000	€ 3.049	[13]
Transplant (procedure)	€ 73,774	€ 62,648	€ 84,900	€ 4.775	[13]
Transplant (following years)	€ 2,365	€ 0	€ 4,729	€ 1.015	[13]
SVR to F0‐F3 states	€ 0	€ 0	€ 0	€ 0	[13]
SVR to ILD states^†^	€ 1,440	€ 397	€ 2,483	€ 448	[13]

	Utilities				
Health state	Base‐case	Min	Max	SE	Source
F0	0.82	0.4	1.0	··	[21]
F1	0.82	0.4	1.0	··	[21]
F2	0.82	0.4	1.0	··	[21]
F3	0.82	0.4	1.0	··	[21]
F4	0.78	0.4	1.0	··	[21]
DC	0.65	0.3	1.0	··	[21]
HCC	0.25	0.1	0.4	··	[21]
Transplant (procedure)	0.5	0.3	0.7	··	[21]
Transplant (following years)	0.7	0.4	1.0	··	[21]
SVR to F0‐F3 states	1	0.5	1.0	··	[21]
SVR to ILD states ^†^	0.91	0.5	1.0	··	[21]

Note

Abbreviations: Ab, Antibodies; Ag, Antigen; DC, Decompensated Cirrhosis; HCC, Hepatocellular Carcinoma; ILD, Irreversible Liver Disease (F4, DC and HCC); RNA, Ribonucleic Acid; SE, Standard Error; SVR, Sustained Virologic Response.

### 2.1 Model structure

**FIGURE 1:**
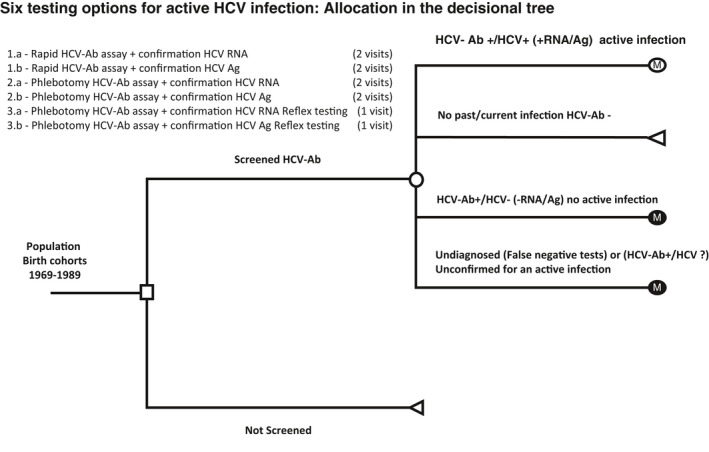
Decision tree model scheme. Option 1: HCV‐Ab by rapid assay on oral or blood specimen followed by ( 1.a) second sample serum/plasma testing for HCV‐RNA or (1b) serum/plasma HCV‐Ag. Option 2: HCV‐Ab by a laboratory‐based assay on serum/plasma followed by (2a) second sample serum/plasma testing for HCV‐RNA or (2b) serum/plasma HCV‐Ag. Option 3: HCV Ab on serum/plasma by a laboratory‐based assay followed by same sample reflex testing for (3a) HCV‐RNA or (3b) HCV‐Ag (3b) on the initial sample. Abbreviations: Ab, Antibodies; Ag, Antigen; HCV, Hepatitis C Virus; RNA, Ribonucleic Acid

The model starts with a decision probabilistic tree that simulates HCV testing in the general population cohort born between years 1969‐1989 with a coverage rate of 70% (approximately 12,000,000 individuals eligible for testing). This first part of the model identified, categorized and evaluated all patients with an active HCV infection that could be potentially diagnosed by each screening option. Each strategy in the decision tree considers the detection of HCV antibodies (HCV‐Ab) results and in case of positive test the confirmation of active infection through HCV‐RNA or HCV core antigen (HCV‐Ag) testing either by conventional two steps or reflex testing (single step). We have considered the following definitions:

1. Active infection is defined as the presence of markers of viral replication in chronic infection state.
2. ‘Reflex testing’ means that HCV‐RNA of HCV‐Ag is performed on the same serological specimen with a positive anti‐HCV finding. If the reflex test is positive, an active HCV infection is diagnosed and the patient will be referred for disease staging and treatment.^9^
3. “Undiagnosed” cases were defined as having active HCV infection but with HCV‐Ab false negative results, or false negative confirmation test following an anti‐HCV positive test result.^17^, ^18^, ^19^, ^20^
4. “Unconfirmed” active infection was defined as HCV‐Ab positive without confirmation of active infection.
5. In both undiagnosed and unconfirmed groups, individuals with active infection will not be linked to care following the first HCV‐Ab test (Figure 1).

**FIGURE 2:**
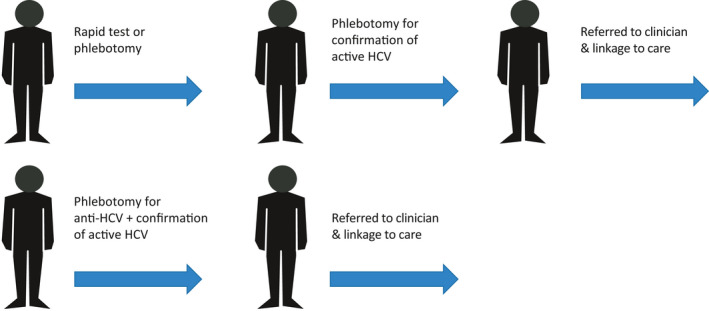
Patient journey to HCV active infection diagnosis. Abbreviations: HCV, Hepatitis C Virus

In the simulations, we considered six alternative diagnostic algorithms (1a to 3b) for diagnosis of active HCV infection. In each scenario, the initial screening is performed through an assay for HCV‐Ab, either rapid or laboratory (phlebotomy) based, followed by confirmation through HCV‐RNA or HCV‐Ag testing (laboratory based). Four of these scenarios require two visits for confirmation of active HCV infection with subsequent patient referral, while the remaining two require only one visit (Figure 2). Based on previous published reports we assumed the drop‐off of patients from the first visit for antibody detection and the second visit for confirmatory testing to be 45% (ranges 36%‐54%).^16^, ^22^

### 2.2 Transition probabilities

Table 1 summarizes the available information regarding the different screening strategies considered in the decision tree. The Markov model considered published transition probabilities previously used in the same modelling (Table S1).^12^, ^13^

Disease progression depends on the linkage to care probabilities^12^, ^13^ and the previously estimated Fibrosis stage (F, graded from 1 to 4) at the time of the diagnosis.^14^ Throughout annual cycles, patients could remain in their current liver disease stage or progress to a worse state according to the natural history of the disease or stopped/slowed down liver disease progression by HCV elimination by treatment (patients with confirmed active infection linked to care and cure). An immediate linkage to care following the diagnosis of an active infection diagnosis and its consequences over a period of 10 years (up to 2030) was assumed for each screening strategy.

Individuals with undiagnosed or unconfirmed infection were considered as not being linked to care. Therefore, these individuals were assumed to either progress according to the natural history of disease until 2030 or diagnosed because of complications of cirrhosis (1 year later for F4 fibrosis stage and 4 years later for F3 fibrosis stage that progress to cirrhosis). Unconfirmed and undiagnosed F0‐F2 fibrosis also progress according to the natural history of the disease until the year 2030. After these periods, the model assumes that all patients were diagnosed and treated. For unscreened individuals, the model assumes progression according to the natural disease history.

### 2.3 Epidemiological and clinical parameters

The model assumes a fibrosis distribution for the diagnosed patients through screening as it is reported in our previous studies, based on infection and disease burdens for yet to be treated patients.^5^, ^14^ For screened but undiagnosed or unconfirmed (see definitions above) individuals the distribution of fibrosis from F0 to F3 (asymptomatic) was made at the time of modelling start and F4 were excluded on the assumption of being diagnosed by screening as the diagnosis should be done due to a symptomatic disease (cirrhosis) (Table 1).

### 2.4 Efficacy: SVR rates

The efficacy of second‐generation DAA regimens used in the Markov Model, was considered 98%^9^ and was stratified by the presence or absence of cirrhosis (F0‐F4, DC, or HCC (Table S2). F0‐F3 does not progress furthermore following viral eradication whereas in F4/DC or HCC a slow progression after HCV eradication was considered.

### 2.5 Cost of screening and HCV health states

The decision tree model considers only the unit price related to the first‐line test (HCV‐Ab), the cost of administration of the first‐line test and the tariff associated with the second line tests (Table 2).

Direct healthcare costs were those associated with DAAs and the management of HCV‐related diseases. The average and range of costs (MIN–MAX) of HCV‐related liver disease were derived from the literature (Table 2).^12^, ^21^ The average treatment cost of DAAs was estimated based on expert opinion and non‐official previously well‐validated sources.^14^ Costs were expressed in Euros and were discounted at a rate of 3% annually.

### 2.6 Model outcomes

Health outcomes were expressed in terms of quality‐adjusted life‐years (QALYs) incorporating mortality and morbidity associated with a particular health state. Utilities were associated with each health state and a full health condition was assumed for undiagnosed individuals. The model considers in the base‐case the same utilities previously reported and used in our modelling studies on HCV treatment (Table 2).^21^QALYs were discounted by an annual rate of 3%.

### 2.7 Incremental cost‐effectiveness analysis

Strategies were compared using incremental cost‐effectiveness ratios (ICERs), calculated as the additional cost divided by the additional health benefit of the alternative with lower QALYs or compared with the Standard of Care (SoC) (Figure 1, testing option 2a), and expressed as the cost per QALY gained. We used the commonly cited Italian willingness‐to‐pay (WTP) threshold of €25,000/QALY.^23^ Outcome measures were assessed from a healthcare sector perspective.

### 2.8 Multivariate sensitivity analysis

We performed deterministic sensitivity analyses (DSA) to identify parameters with the greatest impact on cost‐effectiveness. We considered one‐way variations (Table 1 and 2) and calculated the corresponding ICER estimated for the reflex approach vs the SoC (HCV‐Ab first than confirmatory HCV‐RNA or HCV‐Ag test with two visit approach) for these parameters:

1. Tests sensitivity and specificity
2. Ability on linking to care
3. All HCV tests’ costs (HCV‐Ab, HCV‐RNA and HCV‐Ag costs)
4. Other direct costs (healthcare costs related to chronic liver disease)
5. Health status utilities

Probabilistic Sensitivity Analysis (PSA) was performed considering a gamma distribution for costs and beta distribution for epidemiological parameters.^21^ During PSA, values were varied widely within the ranges stated in Tables 1 and 2, and were randomly sampled from the respective distributions with 5,000 Monte Carlo simulations. Based on these simulations, the cost‐effectiveness acceptability curve (CEAC) for the best cost‐effective scenario vs lower efficacy screening option and second most effective screening alternative were presented.

## 3 RESULTS

The screening costs for different options evaluated varied from €60.5 million to €66.2 million with higher costs in algorithms that contain HCV‐RNA test for confirmation vs the HCV‐Ag (Table 3). An increasing trend of treatment costs on reflex testing vs other options has been estimated, ranging from € 414.1 million to € 464.7 million (Table 3). This reflects the higher number of treated patients according to each screening strategy, being highest in the reflex (3a and 3b) vs the algorithms that require two visits for the detection of HCV active infection.

**TABLE 3:** Base‐case cost results (Italy – assuming a 70% coverage rate)

	Screening cost	Screening administration cost	Treatment cost	Disease cost
1.b ‐ Rapid Ab assay +confirmation (Ag)	€ 60,553,442	€ 35,654,615	€ 414,125,853	€ 319,713,702
1.a ‐ Rapid Ab assay +confirmation (RNA)	€ 64,458,378	€ 35,654,615	€ 418,340,715	€ 321,028,714
2.b ‐ Lab‐based Ab assay +confirmation (Ag) with second sample taken	€ 60,617,239	€ 59,424,358	€ 421,150,624	€ 321,905,388
2.a ‐ Lab‐based Ab assay +confirmation (RNA) with second sample taken	€ 64,733,486	€ 59,424,358	€ 425,365,486	€ 323,220,400
3.b ‐ Lab‐based Ab assay +confirmation (Ag) reflex testing	€ 60,974,498	€ 59,424,358	€ 460,489,341	€ 334,178,830
3.a ‐ Lab‐based Ab assay +confirmation (RNA) reflex testing	€ 66,274,095	€ 59,424,358	€ 464,704,203	€ 335,493,841

Note

Abbreviations: Ab, Antibodies; Ag, Antigen; RNA, Ribonucleic Acid.

The comparison of cost effectiveness results is based considering as reference the option which produce the lowest QALYs. As shown in Table 4, the reference is option 1b (Rapid Ab assays + confirmation HCV‐Ag).

**TABLE 4:** Base‐case cost‐effectiveness

			ICER vs less effective option			ICER Reflex vs SoC		
	Overall Cost	QALYs	Inc QALYs	Inc Cost	ICER	Inc QALYs	Inc Cost	ICER
1.b ‐ Rapid Ab assay +confirmation (Ag)	€ 830,047,612	866,835	–	–	–	–	–	–
1.a ‐ Rapid Ab assay +confirmation (RNA)	€ 839,482,421	875,803	8,969	€ 9,434,809	€ 1,052	–	–	–
2.b ‐ Lab‐based Ab assay +confirmation (Ag) with second sample taken	€ 863,097,608	881,782	14,948	€ 33,049,996	€ 2,211	–	–	–
2.a ‐ Lab‐based Ab assay +confirmation (RNA) with second sample taken (SoC)	€ 872,743,730	890,751	23,916	€ 42,696,118	€ 1,785	–	–	–
3.b ‐ Lab‐based Ab assay +confirmation (Ag) reflex testing	€ 915,067,026	965,489	98,654	€ 85,019,414	€ 862	74,738	€ 42,323,296	€ 566
3.a ‐ Lab‐based Ab assay +confirmation (RNA) reflex testing	€ 925,896,498	974,458	107,623	€ 95,848,886	€ 891	83,707	€ 53,152,768	€ 635

Note

**Abbreviations**: Ab, Antibodies; Ag, Antigen; ICER, Incremental Cost‐Effectiveness Ratio; Inc, Incremental; QALY, Quality‐Adjusted Life Years, RNA, Ribonucleic Acid; SoC, Standard of Care.

All ICERs estimated are far below the WTP threshold. The lowest ICER is estimated for the HCV‐Ag reflex testing, however, in a range of cost effective options (varying from €2,211‐862/QALY) the best option is given by the HCV‐RNA reflex testing in that it produces the highest QALYs (974,458) or the highest incremental QALYs (107,623) vs the reference (1b) option which is the least effective option. Comparing reflex vs two steps diagnostic algorithms a persistent increase in QALYs with a very low ICERs varying from €566‐635 per QALYs is estimated (Table 4).

**FIGURE 3:**
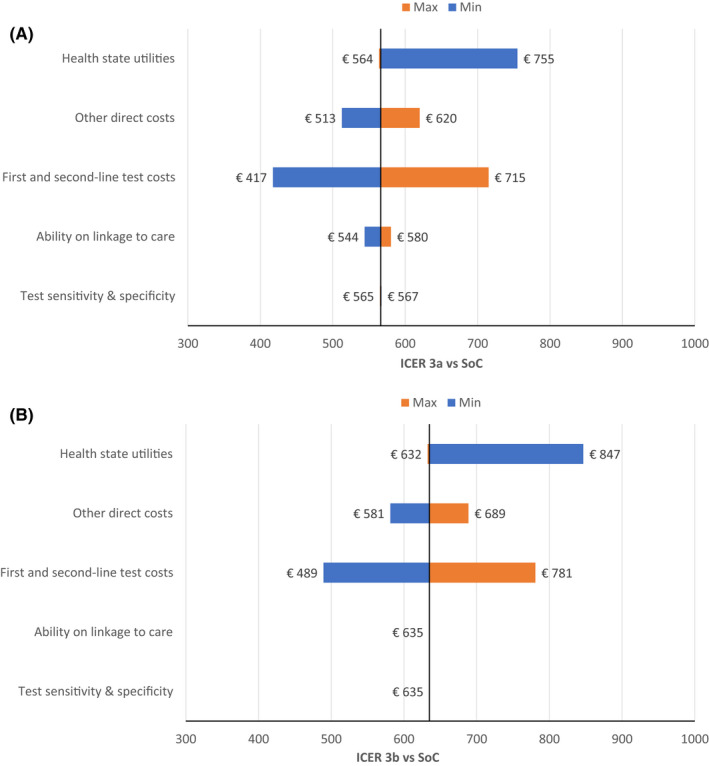
Tornado diagram of A, Lab‐based HCV‐Ab assay +confirmation (HCV‐RNA) reflex testing and B, Lab‐based HCV‐Ab assay +confirmation (HCV‐Ag) reflex testing. Abbreviations: Ab, Antibodies; Ag, Antigen; HCV, Hepatitis C Virus; ICER, Incremental Cost‐Effectiveness Ratio; RNA, Ribonucleic Acid; SoC, Standard of Care

The deterministic sensitivity analysis (Figure 3) shows that the most sensitive parameters of the model are represented by the variation of the utilities associated with the disease states (a 25% variation causes an ICER increase of one third times higher than the base‐case), 1st and 2nd line test costs (25% variation causes ±17% of the ICER value) and other direct costs (±8% of variation for the corresponding ICER).

**FIGURE 4:**
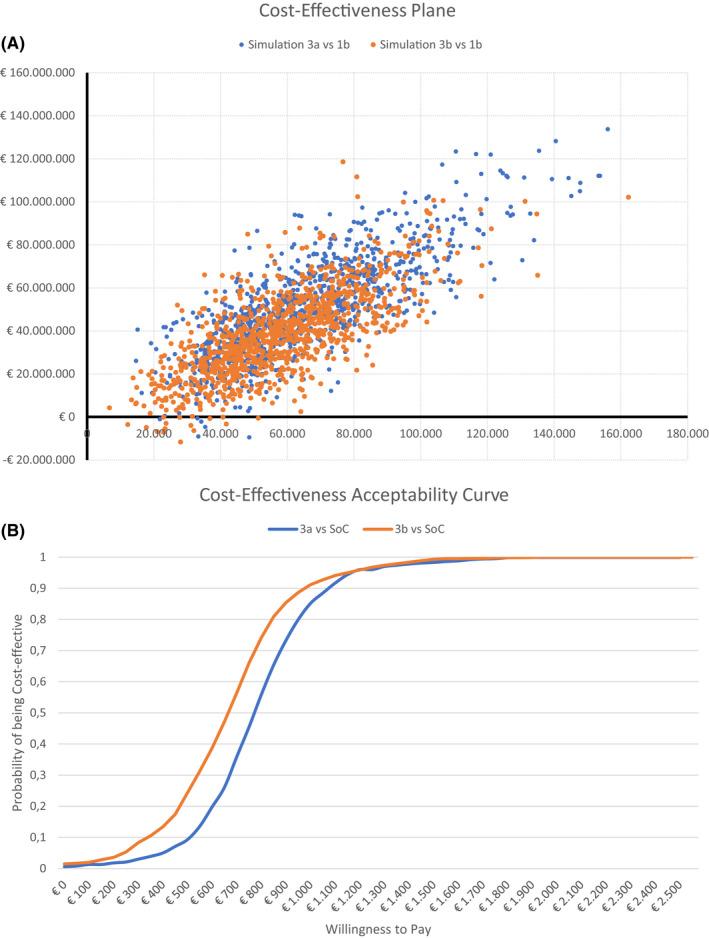
A, Cost‐Effectiveness Acceptability Curve of Lab‐based Ab assay +confirmation (HCV‐RNA or HCV‐Ag) reflex testing and B, Cost‐Effectiveness Plane of Lab‐based HCV‐Ab assay +confirmation (HCV‐RNA or HCV‐Ag) reflex testing vs rapid HCV‐Ab assay +confirmation (HCV‐Ag). Abbreviations: Ab, Antibodies; Ag, Antigen; HCV, Hepatitis C Virus; RNA, Ribonucleic Acid; SoC, Standard of Care

Probabilistic sensitivity analysis confirmed that the reflex approach compared to the SoC would be cost‐effective for >90% of simulations at a minimum WTP threshold of €1,000/QALY gained and for >99.9% of simulation at a maximum WTP threshold of €25,000/QALY gained (Figure 4A). Most points on the cost‐effectiveness plane (Figure 4B) are distributed in the northeast quadrant, showing that reflex approach was associated with higher costs and greater benefits than SoC. In some cases, reflex is dominant if compared with SoC (<5% of the simulation in the southeast quadrant).

## 4 DISCUSSION

In the general population, only 40%‐70% of HCV‐Ab positive individuals have an active infection,^15^ requiring confirmation by HCV‐RNA or HCV‐Ag. To date in Italy and other developed countries, traditionally most diagnostic settings use a two‐step (two visits) approach including a phlebotomy antibody test in step one and HCV‐RNA confirmation in step two. Traditional approaches that include several steps in the diagnostic process often lead to incomplete diagnosis.^8^, ^22^

This is the first economic analysis that evaluates the cost effectiveness profile of different diagnostic strategies aimed at maximising HCV elimination efforts. The key finding of our study is that reflex testing, even if more expensive in terms of screening and overall treatment costs, also achieved the highest number of confirmed infections and therefore associated QALYs (health benefit) compared to two step testing strategies within a 10 year time horizon (up to 2030).

Importantly these results suggest that despite the highest screening cost, HCV‐RNA reflex is the most cost effective strategy. Our findings suggest that the reflex testing should become the standard of care for the diagnosis of active HCV infection in both public and private laboratories. From a public health perspective, the real‐world utility of HCV reflex testing at population level is also demonstrated in a recent cost‐effectiveness analysis on routine universal HCV screening in urban emergency departments in England.^24^

In Italy, based on WHO and EASL recommendations reflex testing is already explicitly included in the recommendations of Italian Ministry of Health free of charge screening law decree.^7^, ^25^ However, its implementation at Regional level has not yet been realized. The findings of this study will serve as evidence to increase the awareness of regional stakeholders, including medical and non‐medical staff involved in the screening process to optimize the patient pathways for active HCV infection.

Our study has been tailored to the Italian context but the reflex testing strategies in detecting the active infection has proven effective also in other countries.^24^, ^25^, ^26^, ^27^, ^28^ We believe that the structure and findings of our model are well‐suited for other countries who have ongoing or soon to be started high volume testing programmes to address HCV elimination as defined by the WHO.^1^ Without an active screening there will be a progressive decrease in diagnosed cases and few countries may be able to reach the mortality reduction endpoint.^6^, ^29^, ^30^ Broader screening for HCV would likely be cost‐effective, but significantly reducing HCV‐related morbidity and mortality would also require improved rates of referral, treatment and cure and this care cascade starts with maximising the detection of active infection. Reflex testing, as outlined in our evaluation and in line with other recommendations,^9^, ^10^, ^11^ promises to be the most cost effective screening algorithm in terms of maximizing detection of active infection as first step into the HCV care cascade.

Within the two reflex testing strategies the use of HCV‐Ag assay is not widespread in Italy, however, we feel that its inclusion in our cost‐effectiveness analysis is justified for completeness, especially as our findings suggest that reflex testing with HCV‐Ag compared to conventional two step testing could prove useful for high volume screening in countries where HCV‐RNA testing is not available or affordable.^9^

In some settings HCV‐Ab point of care testing has been shown to reduce time between the initial observation and treatment administration.^9^ We have demonstrated that this approach, though attractive for outreach screening for special populations, subsequently requires referral for conventional phlebotomy confirmation of active infection and was the less cost‐effective option out of the six screening pathways analysed.

Health state utilities, costs of screening tests and direct medical costs had the highest impact on the ICER of reflex testing compared to the (two step) SoC. However, the highest ICER (resulting from the lowest reported utilities) was less than €800/QALY, far lower than the WTP threshold. Based on the simultaneous variations of the parameters evaluated by the PSA analysis, reflex testing although more expensive than the two steps testing, remained highly cost effective in 99.9% of the scenarios and dominant in 5%, which indicates less costs sustained and higher efficacy for reflex vs the two step SoC diagnostic algorithm.

The strength of our study is that using a specifically designed Markov model of liver disease progression enables the evaluation of the relative costs of screening, treatment and overall disease costs, which change according to the proportion of undiagnosed patients assumed, based on data from real life studies.^16^ Specifically, in a prospective study a substantial drop off rate of 45% occurred at the second visit for confirmatory serum/plasma testing, when the two steps diagnostic algorithm was applied.^16^ In contrast, the introduction of reflex testing and warning messages for referral resulted in reduction in drop‐off rate to just 17% with associated increase in the number of patients diagnosed with active infection and therefore evaluated for viral eradication by antiviral treatment. Other studies reported that in the DAA era, 27%‐68% of infected patients reached the stage of confirmed infection.^31^, ^32^ Based on these data, in our analysis we assumed an increase of the proportion of individuals with the diagnosis of active infection by reflex testing as compared with the two steps approach.

The first and main limitation of this study is the assumption for drop‐off rate for each scenario. Reports are scarce, therefore in the absence of Italian data we had to extrapolate from recent estimates reported in the literature.^16^, ^22^, ^31^ Further in our analysis we considered 70% uptake for HCV screening as it was reported for other screening campaigns in Italy. As we examined a general population birth cohort (not more challenging specialist populations) we feel that this uptake assumption is justified. We assumed immediate linkage to care following the active infection diagnosis for the 10 year period (up to 2030). Even if optimistic, this could be achievable within the traditional healthcare system and serves to highlight the need for continuous improvement of the entire patient pathway. Finally, we considered only direct diagnostic assay costs for our study. However, the reduction of healthcare visits and associated additional staff and patients costs would likely further increase the cost‐effectiveness estimates of the reflex testing strategy.

These screening algorithms are suggested only for general population unaware of chronic HCV infection. As the frequency of acute infection in general population without apparent risk factors is likely very low at population level in Italy and other developed countries, we did not consider diagnosis of acute infection in this evaluation. Different diagnostic algorithms should also be evaluated for marginalized populations.

In conclusion, our findings suggest same sample reflex testing, using either HCV‐RNA or HCV‐Ag, is the most cost effective diagnostic algorithm for countries wanting to embark on high volume HCV testing. Our data confirm the EASL and WHO guidelines recommending reflex testing as best practice in identifying HCV active infection in general population as compared to the other screening approaches. In line with the Italian Ministry of Health HCV screening recommendation, reflex testing should be adopted by the regional Italian health authorities as standard of care for the HCV diagnostic pathway.

## CONFLICT OF INTEREST

Andrea Marcellusi has nothing to disclose; Francesco S. Mennini received advisor/speaker/research grants from Abbvie, MSD, Gilead, BMS; Maurizia R Brunetto received advisor/Speaker grants from AbbVie, Gilead, Janssen, Roche, EISAI‐MSD and is Coordinator of the control room for the application of the Resolution n.397 April 2018 of the Tuscany Government for the control of chronic hepatitis C; Loreta A. Kondili received Speaker grants from Abbvie and Gilead. Murad Ruf is an employee of Gilead. Claudio Galli is employed by and owns stocks from Abbott.

## GUARANTOR OF THE ARTICLE

The corresponding author had full access to all the data in the study and had final responsibility for the decision to submit for publication.

## Supporting information

Supplementary Material

## Data Availability

Data sharing is not applicable as no new data were generated/ the article describes entirely theoretical research.
