# Supplementary material for: Optimizing diagnostic algorithms to advance Hepatitis C elimination in Italy: A cost effectiveness evaluation
Source: Liver Int. 2021 Oct 8;42(1):26–37. doi: 10.1111/liv.15070 (PMC9292516; doi:10.1111/liv.15070)
Supplement: Supplementary file 1 — Supplementary Material [file LIV-42-26-s001.docx]

**Optimizing diagnostic algorithms to advance HCV elimination in Italy: A cost effectiveness evaluation**

***Supplementary Material***

Andrea Marcellusi PhD^1,2^, Francesco Saverio Mennini^1,2^, Murad Ruf MD^3^, Claudio Galli PhD^4^, Alessio Aghemo MD^5^, Maurizia R. Brunetto MD^6^, Sergio Babudieri MD^7^, Antonio Craxi MD^8^, Massimo Andreoni MD^9^, Loreta A. Kondili MD PhD^10^

Correspondence to:

Dr. L.A. Kondili, Center for Global Health, Istituto Superiore di Sanità, Viale Regina Elena 299, 00161 Rome, Italy. Tel: +39 0649903813/ [+39 3473143971](tel:%2B39%203473143971). Fax: +39 0649902012

E-mail: loreta.kondili@iss.it

**Figure S1 – Natural History of Hepatitis C Virus (HCV) Markov Model [1-3]**


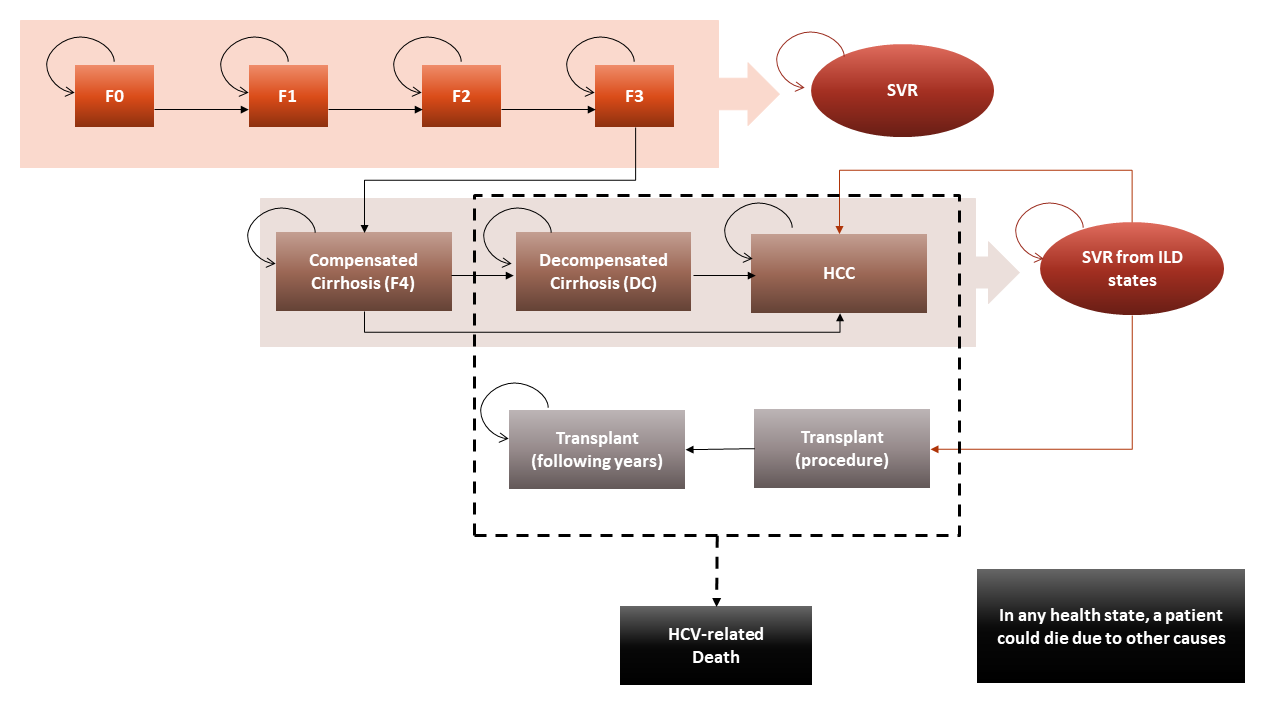


**Legend**: fibrosis stage; SVR: sustained virologic response; HCV: hepatitis C virus; DC: decompensated cirrhosis; HCC: hepatocellular carcinoma; ILD: irreversible liver damage

The model structure considers 13 disease states (fibrosis stages from F0 to F4, DC, hepatocarcinoma (HCC), first-year transplant and subsequent years transplant, sustained virologic response (SVR) from F0 to F3, SVR from irreversible liver damage (ILD), HCV-related death, and death from other causes) and 41 transition probabilities. Events constituting advanced liver disease, such as ILD or DC, were considered as cumulative events in the model and not mutually exclusive.

Progression of HCV liver disease was considered to increase with the severity of liver fibrosis (from F0 to F4 according to the Metavir classification) or progression to ILD stages.

For the F0-F4 and DC disease states, the probabilities of achieving SVR, disease progression, and HCV-related death were estimated.

**Table S1 – Transition probabilities and efficacy of treatment (Base-case and probabilistic sensitivity analysis parameters)*.***

| **Annual probability of disease progression** | **Base-case** |  | Standard Error included in the PSA | **Source** |
| --- | --- | --- | --- | --- |
| F0 to F1 | 0.117 |  | 0,010 | [4] |
| F1 to F2 | 0.085 |  | 0,007 | “ |
| F2 to F3 | 0.120 |  | 0,010 | “ |
| F3 to F4 | 0.100 |  | 0,009 | [5] |
| F4 to Decompensated Cirrhosis (DC) | 0.030 |  | 0,003 | “ |
| F4 to HCC | 0.050 |  | 0,004 | “ |
| Decompensated Cirrhosis to HCC | 0.100 |  | 0,009 | “ |
| Decompensated Cirrhosis to Transplant | 0.110 |  | 0,009 | “ |
| HCC to Transplant | 0.200 |  | 0,017 | [6] |
| SVR to HCC ^†^ | 0.008 |  | 0,000 | Assumption from [7] |
| SVR to Transplant^†^ | 0.016 |  | 0,002 | Assumption from [7] |
| **Annual probability of progressing to death** | **Base-case** |  |  | **Source** |
| Decompensated Cirrhosis to Death (liver-related) | 0.090 |  | 0,008 | [7] |
| HCC to Death (liver-related) | 0.430 |  | 0,037 | [6] |
| Transplant (procedure) to Death (liver-related) | 0.150 |  | 0,013 | “ |
| Transplant (following years) to Death (liver-related) | 0.057 |  | 0,005 | “ |
| Death from all other causes Italy | 0.062 |  |  | Average age 60.17 [8] |
| **Efficacy of treatments – 2017-2019** | **Base-case** |  |  | **Source** |
| F0-F3 to SVR (Genotype 1) | 0.980 |  |  | Table A2 and A3 |
| F4-DC to SVR (Genotype 1) | 0.931 |  |  | “ |
| F0-F3 to SVR (Genotype 2) | 0.980 |  |  | “ |
| F4-DC to SVR (Genotype 2) | 0.980 |  |  | “ |
| F0-F3 to SVR (Genotype 3) | 0.950 |  |  | “ |
| F4-DC to SVR (Genotype 3) | 0.884 |  |  | “ |
| F0-F3 to SVR (Genotype 4 and more) | 0.980 |  |  | “ |
| F4-DC to SVR (Genotype 4 and more) | 0.961 |  |  | “ |

**Table S2 - SVR rates by HCV RNA genotype and fibrosis stage used for estimating mean rates of SVR in 2017-2019**

| **Genotype** | **SVR for F0-F3*** | **SVR for F4*** | **SVR for DC*** | **References** |
| --- | --- | --- | --- | --- |
| Genotype 1 | 0.98 | 0.94 | 0.88 | [9] |
| Genotype 2 | 0.98 | 0.99 | 0.85 | [9] |
| Genotype 3 | 0.95 | 0.89 | 0.85 | [9] |
| Genotype 4 and ohters | 0.97 | 0.98 | 0.88 | [9] |

*mean of treatments commonly used in Italy in 2019

**Table S3 – Genotype and fibrosis stage distribution in Italy**

| **Genotype Distribution Italy** | **Percentage distribution** | **Source** |
| --- | --- | --- |
| G1 | 58% |  |
| G2 | 21% | **[10]** |
| G3 | 14% |  |
| G4 and other | 8% |  |
| **Fibrosis Distribution Italy** | **Percentage distribution** | **Source** |
| F0 – F2 | 62% |  |
| F3 | 16% |  |
| F4 | 19% | **[10]** |
| DC | 2% |  |
| HCC | 1% |  |

**Table S4 – Estimated screening cost per diagnosed patient**

|  | **Diagnosed** | **Screening Cost** | **Cost per Diagnosed pts** |
| --- | --- | --- | --- |
| 1.b - Rapid Ab assay + confirmation (Ag) | 35.823 | € 60.553.442 | € 1.690 |
| 1.a - Rapid Ab assay + confirmation (RNA) | 38.238 | € 64.458.378 | € 1.686 |
| 2.b - Lab-based Ab assay + confirmation (Ag) with second sample taken | 39.848 | € 60.617.239 | € 1.521 |
| 2.a - Lab-based Ab assay + confirmation (RNA) with second sample taken | 42.263 | € 64.733.486 | € 1.532 |
| 3.b - Lab-based Ab assay + confirmation (Ag) reflex testing | 62.388 | € 60.974.498 | € 977 |
| 3.a - Lab-based Ab assay + confirmation (RNA) reflex testing | 64.803 | € 66.274.095 | € 1.023 |

**References**

1. Mennini, F.S., et al., The impact of direct acting antivirals on hepatitis C virus disease burden and associated costs in four european countries. Liver Int, 2021.

2. Marcellusi, A., et al., Economic Consequences of Investing in Anti-HCV Antiviral Treatment from the Italian NHS Perspective: A Real-World-Based Analysis of PITER Data. Pharmacoeconomics, 2019. 37(2): p. 255-266.

3. Marcellusi, A., et al., Early Treatment in HCV: Is it a Cost-Utility Option from the Italian Perspective? Clin Drug Investig, 2016. 36(8): p. 661-72.

4. Thein, H.H., et al., Estimation of stage-specific fibrosis progression rates in chronic hepatitis C virus infection: a meta-analysis and meta-regression. Hepatology, 2008. 48(2): p. 418-31.

5. Dienstag, J.L., et al., A prospective study of the rate of progression in compensated, histologically advanced chronic hepatitis C. Hepatology, 2011. 54(2): p. 396-405.

6. Wright, M., et al., Health benefits of antiviral therapy for mild chronic hepatitis C: randomised controlled trial and economic evaluation. Health Technol Assess, 2006. 10(21): p. 1-113, iii.

7. Morgan, R.L., et al., Eradication of hepatitis C virus infection and the development of hepatocellular carcinoma: a meta-analysis of observational studies. Ann Intern Med, 2013. 158(5 Pt 1): p. 329-37.

8. EUROSTAT (2018). Lifetable from European Countries. (Available <http://appsso.eurostat.ec.europa.eu/nui/show.do> last Access 04/2020).

9. Kondili, L.A., et al., Modeling cost-effectiveness and health gains of a "universal" versus "prioritized" hepatitis C virus treatment policy in a real-life cohort. Hepatology, 2017. 66(6): p. 1814-1825.

10. Monitoraggio, U.R.d. Aggiornamentio dati Registri AIFA DAAs, epatitie C cronica. 2019, Agenzia Italiano del Farmaco.<http://www.agenziafarmaco.gov.it/content/registri-farmaci-sottoposti-monitoraggio>. .
